# Benthic studies adjacent to Sakhalin Island, Russia, 2015 II: energy content of the zoobenthos in western gray whale feeding grounds

**DOI:** 10.1007/s10661-022-10020-z

**Published:** 2022-10-18

**Authors:** Jennifer L. Maresh, Arny L. Blanchard, Natalia L. Demchenko, Ilya Shcherbakov, Lisanne Aerts, Lisa K. Schwarz

**Affiliations:** 1 Department of Biology, West Chester University, West Chester, PA 19383 USA; 2 Blanchard Ecological, AK 99705 North Pole, USA; 3 A.V. Zhirmunsky National Science Center of Marine Biology, Far East Branch of Russian Academy of Sciences, Vladivostok, Russia; 4 LAMA Ecological, Anchorage, AK 99502 USA; 5 Ocean Sciences and Institute of Marine Sciences, University of California, Santa Cruz, CA 95060 USA

**Keywords:** Amphipods, Calorimetry, *Eschrichtius robustus*, Gray whale prey, Macrobenthos, Russia, Sakhalin Island, Sea of Okhotsk

## Abstract

The waters adjacent to the northeastern coast of Sakhalin Island, Russia, are an important feeding ground for the endangered western gray whale. Data on the energy available to foraging whales from their prey resources is required for researchers interested in modeling the bioenergetics of whale foraging, but little energy content information is available for the benthic prey communities of gray whales in this region. In this study, we describe the energy density (ED), biomass, and total energy availability (ED × biomass) of benthic prey sampled from two gray whale foraging areas adjacent to Sakhalin Island: the nearshore and offshore feeding areas. ED varied almost seven-fold among benthic taxa, ranging from 1.11 to 7.62 kJ/g wet mass. Although there was considerable variation within most prey groups, amphipods had the highest mean ED of all of groups examined (5.58 ± 1.44 kJ/g wet mass). Small sample sizes precluded us from detecting any seasonal or spatial differences in mean ED within or among taxa; however, mean biomass in the offshore feeding area was, in some cases, an order of magnitude higher than mean estimates in the nearshore feeding area, resulting in higher mean total energy available to foraging gray whales offshore (958–3313 kJ/m^2^) compared to nearshore (223–495 kJ/m^2^). While the proportion of total energy accounted for by amphipods was variable, this prey group generally made up a higher proportion of the total energy available in the benthos of the offshore feeding area than in the benthos of the nearshore feeding area. Data presented here will be used to inform bioenergetics modeling of the vital rates of mature females in an effort to improve understanding of population growth limits for western gray whales.

**Supplementary information:**

The online version contains supplementary material available at 10.1007/s10661-022-10020-z.

## Introduction

**Fig. 1:**
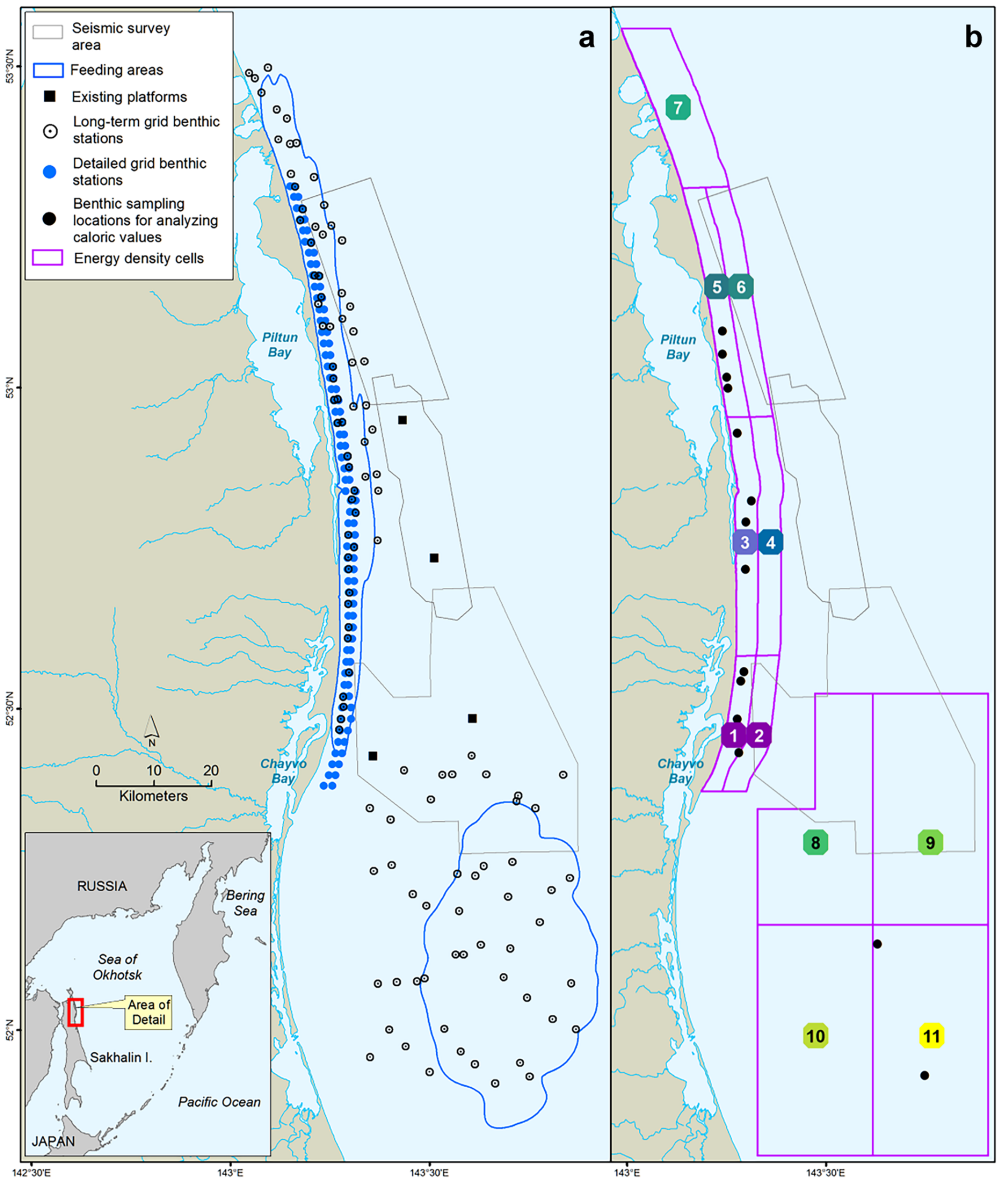
Map showing the 2015 seismic survey areas (gray polygons) and their proximity to western gray whale feeding areas (blue polygons in **a**) derived from 2001–2014 shore-based, vessel-based, and aerial survey programs. **b** The locations where caloric samples were taken, and the 11 cells for which energy density estimates were calculated (purple polygons). In 2015, the detailed benthic sampling occurred in cells 1, 3, and 5, and sampling in other cells was less dense

While eastern gray whales (*Eschrichtius robustus*) are believed to be approaching carrying capacity, western (Korean-Okhotsk) gray whales have been slow to recover since the end of commercial whaling in the 1970s (Cooke et al., 2018). The northeastern coast of Sakhalin Island within the Sea of Okhotsk is known as an important summer feeding area for the endangered whales, where a sizeable portion of the population returns after a breeding season spent at lower latitudes (Blokhin et al., 1985; Meier et al., 2007; Tyurneva et al., 2017; Weller et al., 1999; Yakovlev et al., 2009). Aerial surveys have revealed two distinct feeding areas based on the densities of foraging whales: a nearshore area adjacent to the Piltun and Chayvo Bays, and an offshore area 30–45 km from the Sakhalin coast (Fig. 1a) (Demchenko et al., 2016; Meier et al., 2007). These areas are within the region of highest primary and secondary production in the Okhotsk Sea and are considered to be critically important for western gray whales of both sexes and multiple age-classes, including reproductive females and their calves (Bradford et al., 2012; Demchenko et al., 2016).

Over the past two decades, concern for these endangered whales has prompted long-term studies of their ecology, prey communities, and acoustic environments to better understand potential food limitation on population growth, anthropogenic impacts, and mitigation effectiveness (e.g., Bradford et al., 2012; Bröker et al., 2015; Demchenko, 2007, 2010; Demchenko et al., 2016; Durkina et al., 2018; Fadeev, 2013; Gailey et al., 2007, 2011, 2016; Johnson et al., 2007; Kriksunov et al., 2016; Tyurneva et al., 2010, 2017; Vladimirov et al., 2011; Yakovlev et al., 2009). Increased seismic survey activities along the Sakhalin coast in 2015 prompted the development of additional research that, unlike previous work, focused on determining whether observed gray whale behavioral responses—avoiding areas of overlap between anthropogenic activities and essential habitat—could impact vital rates on a population level (e.g., Aerts et al., 2022). This determination requires information on the energy value of gray whale prey in this region.

Like their eastern counterparts, western gray whales are unique among large cetaceans in their feeding behaviors, primarily targeting benthic fauna such as amphipods, mysids, cumaceans, isopods, polychaete worms, and other benthic and epibenthic organisms when they occur in high densities (Darling et al., 1998; Dunham & Duffus, 2002; Fadeev, 2006; Highsmith & Coyle, 1992; Kim & Oliver, 1989; Nerini, 1984). Dense aggregations of energy-rich amphipods such as *Ampelisca eschrichtii* and *Monoporeia affinis* numerically dominate macrobenthic communities on the northeastern Sakhalin Island Shelf and are among the most important prey of western gray whales (Demchenko, 2007, 2010; Demchenko et al., 2016); however, the degree to which the zoobenthos here meet the whales’ energy requirements remains unclear.

While energy content information is available for amphipods and other benthic invertebrate prey of eastern gray whales foraging in the northeastern Bering and southeastern Chukchi Seas (Grebmeier et al., 2006; Highsmith & Coyle, 1990, 1992; Hondolero et al., 2012; Tu et al., 2015; Wilt et al., 2014), very little energy content information has been available for the benthic prey communities in the two known feeding areas of western gray whales along the Sakhalin coast. In the current study, we report the energy content of the main prey species targeted by western gray whales in this region. In addition, we combine energy content with detailed prey biomass data (Blanchard et al., 2022) to characterize the prey-energy available to whales foraging here. Our findings establish an important link between energy requirements and energy availability that may shed light on the dynamics limiting population growth in western gray whales.

## Materials and methods

### Sample collection

From 2001 to 2014, survey programs along the northeastern Sakhalin Island shelf have shown that gray whales feed in close proximity to shore throughout the summer foraging season (Jun–Oct), with increasing numbers found foraging in a second, offshore site as the season progresses (blue polygons in Fig. 1a) (Aerts et al., 2022; Meier et al., 2007). These two distinct feeding areas will hereafter be referred to as the nearshore (~ 600 km^2^, < 20 m depth) and offshore (~ 1700 km^2^, 30–65 m depth) sites. Benthic sampling in the region has also been ongoing since 2001 (open black circles in Fig. 1a) to document benthic community characteristics and gray whale prey dynamics, including the biomass, distribution, and caloric content of the zoobenthos.

In 2015, benthic sampling was greatly expanded to provide higher temporal and spatial resolution of prey biomass within a detailed sampling grid as described in Blanchard et al. (2022; blue circles in Fig. 1). In conjunction with this expanded effort, additional benthic sampling occurred at 13 distinct stations for the purpose of determining the energy density (i.e., caloric content) of prey species (this study; filled black circles in Fig. 1). These stations were chosen non-randomly for additional benthic sampling based on their putative importance to foraging whales (e.g., high whale density, high amphipod biomass), and thus were mostly located close to shore within the nearshore feeding area. Benthic samples were collected at three time intervals: (1) early season, when whale abundance is historically low (late Jun); (2) mid-season, when whale abundance in the nearshore feeding area peaks (mid-Aug); and (3) late season, when gray whale numbers are declining in the nearshore feeding area and generally increasing in the offshore feeding area (mid-Sep and mid-to-late Oct).

To allow for a more spatially detailed analysis, the nearshore and offshore areas were divided into 11 cells, with the nearshore areas able to be divided into smaller cells due to denser benthic sampling. The boundaries of these cells represent differences in acoustic exposure, changes in benthic biomass, and the probability of whale presence. Specifically, and to be consistent with other studies, we divided the nearshore feeding area into seven cells (Fig. 1b). Latitudinal limits defining south (cells 1 and 2), middle (cells 3 and 4), high (cells 5 and 6), and high north (cell 7) cells were roughly based on the three seismic survey project areas (gray polygons in Fig. 1). The eastern boundaries of the three nearshore cells (cells 1, 3, and 5 extending 0–4 km from shore) were set to match those of the detailed benthic prey sampling grid (Blanchard et al., 2019) and represent the area in which whale density is highest (Meier et al., 2007; Vladimirov et al., 2011). The eastern boundaries of the remaining nearshore cells (cells 2, 4, and 6 extending 4–8 km from shore, and cell 7 from 0 to 8 km) were based on the long-term benthic sampling grid (Blanchard et al., 2019). The offshore feeding area, though larger than the nearshore feeding area, was divided into only four cells, because of the less dense benthic sampling grid (Fig. 1b). The boundaries of the four offshore cells (cells 8–11) were based on a combination of the historic benthic sampling grid and whale sightings in the area over time.

As described above, the majority of stations for collection of extra benthic samples for determination of caloric content were located close to shore—these locations correspond with the three coastal nearshore cells 1, 3, and 5. Due to the importance of these sites to foraging whales, benthic samples for caloric content were collected from cells 1, 3, and 5 during all three defined periods (early, mid, and late seasons). Benthic samples for caloric content were collected from the offshore feeding area during the late season only, when whale abundances began to increase offshore.

Benthic samples were collected aboard the R/V *Igor Maksimov* using a van Veen grab with a surface area of 0.2 m^2^. Onboard the vessel, benthic sediments were rinsed over a series of nested sieves with 5.0-, 1.0-, and 0.5-mm mesh screens, and prey samples for caloric analysis were stored without formalin at temperatures of 0 °C or below. Samples were identified to the lowest taxonomic level possible and stored at − 20 °C at the Laboratory of Dynamics of Marine Ecosystems, National Scientific Center of Marine Biology, Far Eastern Branch, Russian Academy of Sciences*.* Frozen samples were later shipped to the University of California, Santa Cruz, USA, and stored at − 20 °C until determination of energy density.

### Energy density determination

In preparation for energy value determination, prey samples were removed from the freezer and thawed at room temperature. Immediately after thawing, samples were rinsed with deionized water, patted dry to remove excess liquid, weighed (± 0.1 mg), and placed in glass scintillation vials. Each sample was homogenized within the vial using dissecting scissors and a spatula-like scoop until a smooth consistency was achieved. Samples were re-weighed to account for any mass lost during homogenization. Homogenized samples were freeze-dried for a minimum of 48 h and re-weighed to calculate the percentage water present in each sample. Calcium carbonate shells (e.g., bivalves) that could not be removed manually from the samples were dissolved by adding 1 M HCl until the sample ceased bubbling (Carabel et al., 2006; Pinnegar & Polunin, 1999). These samples were set aside for 3 h and subsequently freeze-dried for an additional 48 h. Freeze-dried samples were stored in a desiccator until determination of gross energy density.

Gross energy density of each homogenized sample was determined using oxygen bomb calorimetry (e.g., Lenky et al., 2011; Tu et al., 2015). Samples were pelleted (mean: 0.6 g; range: 0.5–1.1 g) and combusted in a Parr 1341 oxygen bomb calorimeter coupled with a Parr 6772 calorimetric thermometer. The calorimeter was calibrated using the gross energy density of 1.0 g of benzoic acid. Standardization tests (*n* = 10) were conducted before running the first prey samples and once again after every 10th prey sample.

Each homogenized species sample was run in triplicate whenever possible. If replicate measurements were not within 2% of the average energy output for a specific sample, a fourth replicate was run if enough species material was available. Only standardization runs reporting gross energy densities within less than 2% of the actual value were retained. Acid titration was not performed after combustion because prior testing determined the variability in gross energy density was less than 0.5% between samples that were titrated compared with those that were not.

When biomass of a specific prey item in a grab sample was large enough to obtain material for three replicate runs of the same species, species samples were analyzed separately for each grab sample. When biomass of a specific prey item in a grab sample was not large enough to obtain material for three replicate runs, species group homogenates were combined by aggregating samples among grabs taken in the same cell during the same period. If further combinations were necessary, samples were combined at the cell level, thereby losing spatial variation but retaining the time stamps of early, mid, and late seasons. If the species mass in the aggregate samples was still small, the samples were additionally combined by sampling period. When there was not enough material of a species to analyze caloric content, results were reported at the order level.

Energy contents of prey taxa were converted from cal g^−1^ dry mass (DM) to kJ g^−1^ wet mass (WM) using the water content of each sample. This was done because conversion to benthic energy available to gray whales requires multiplying energy content by measured biomass per area, which is measured in wet mass. In addition, gray whales consume whole prey, so wet mass is a more appropriate measure for further analyses of gray whale foraging. Shell mass was included in wet mass for bivalves, since gray whales consume entire animals; however, the energy content of bivalve soft tissue alone is also reported.

Due to small sample sizes as well as the unbalanced nature of the dataset, statistical tests for differences in energy density between prey groups, across sampling periods, and across locations were not performed; instead, means and standard deviations are reported for each prey group. For the two prey groups for which a sufficient number of samples were obtained—amphipods and bivalves—means and 95% confidence intervals (Di Stefano, 2004) across seasons and locations are shown.

### Total energy availability

We calculated the total energy available across seasons for gray whales feeding nearshore and offshore of Sakhalin Island in 2015 from six primary prey species groups: Amphipoda, Bivalvia, Cumacea, Isopoda, Polychaeta, and Actinopterygii. To determine differences in total energy availability across sampling seasons and foraging locations, the prey-energy densities from the current study were combined with the measures of prey biomass simultaneously collected in 2015 (Blanchard et al., 2019). As described above, the current study sampled on smaller temporal and spatial scales relative to other concurrent studies in the area, which were sampling over a study area that covered the boundaries of previous years. This means our dataset of zoobenthic energy content covered only a subset of the total spatial area covered by the prey biomass dataset of Blanchard et al. (2019). Thus, our combined study area includes some areas that we did not sample for determination of zoobenthic energy content directly, but that have been sampled for biomass in previous years. In these cases, we assumed prey species from an unmeasured area had similar energy densities as those measured in nearby areas.

Given the low temporal resolution of samples, we pooled biomass over all time periods for the nearshore feeding area cells > 4 km from shore (cells 4, 6, and 7) but retained spatial differences. There were no biomass data available for cell 2 in 2015 because the benthic sampling grid and detailed sampling stations do not overlap with this cell, so it was removed from further analyses.

Because sample size was small for many non-amphipod species groups in this region, additional energy density data were pulled from the literature (Supplementary Table S1) (Hondolero et al., 2012; Stoker, 1978; Tu et al., 2015; Wilt et al., 2014). Some literature provided dry mass values, so energy density was converted to wet mass values using the proportion of solid material (non-water) of each species group, derived as a beta distribution with calculated mean and variance using data from additional literature (Brawn et al., 1968; Tu et al., 2015). Some studies provided point estimates of energy content while other studies provided distributions with means and standard deviations.

To incorporate additional energy content data with our results, we used a Monte Carlo approach and then found the best-fitting distribution to define energy content by species group. To give equal weight to each study, point data were repeated 20,000 times, while mean and standard deviations were used to sample 20,000 times from a normal distribution when such statistics were available. A normal distribution was used because the shape of the distribution was not provided in the literature, and we assumed the distribution followed the most common pattern in nature (Frank, 2009 and citations therein). The best-fitting distribution of energy density was determined using the minimum Cohen *d* value (Supplementary Table S2). A sample of 20,000 provided a smooth distribution of data points that had variability as well as creating a large enough distribution of data points for Cohen’s *d* analysis. Species group biomass values were multiplied by samples from the distributions of energy density to convert to energy content per square meter. For each prey species group, biomass samples were separated into spatial cells and periods in the coastal nearshore cells, while energy density distributions did not vary by cell or period. We report total prey-energy available per square meter and the proportion of that energy attributed to amphipods by sampling period and cell.

## Results

### Energy density determination

Estimates of gross energy density were obtained from a total of 43 prey homogenates encompassing 11 broad taxonomic groups, including five orders of crustaceans and one species of bony fish (Pacific sand lance, *Ammodytes hexapterus*; Table 1). Not all taxa were present across foraging cells or sampling periods within the benthic grabs—for example, all *Actiniaria* spp. were collected from the offshore area during the late season, while all *A. hexapterus* were collected from within the nearshore feeding area, mostly during the mid-season (Table 1). However, the unbalanced representation of prey taxa across locations and seasons may be an artifact of the non-random sampling design as benthic grabs for caloric analysis were specifically targeting locations with high amphipod biomass.

**Table 1:** Energy density (kJ g^−1^ wet mass ± SD) of gray whale prey species collected from NE Sakhalin Island in 2015. Prey species are described according to the lowest taxonomic level of identification; multiple identifications are given when a homogenate for calorimetry consisted of more than one species. “Cell” is the numeric code for defined areas where samples were collected: south (cell 1), middle (cell 3), or north (cell 5) in the nearshore feeding area, or the offshore feeding area (cell 11). “Season” refers to the three time intervals when samples were collected: late-June (early), mid-August (mid), and mid-September through mid-October (late). Samples were pooled across grabs within the same taxon-cell-season combination as described in the text; cases where multiple sets of values are listed for the same taxon-cell-season combination indicate sufficient sample to keep the grabs separate. “All” samples do not include an equal distribution of prey samples from all three nearshore cells and should not be considered a representation of general temporal trends in all areas. The SD represents the standard deviation for replicate measurements of each sample, and data without SD values indicate that the sample was too small for replicate measurements. For bivalves, values that include the whole animal (i.e., with the shell) are included in parentheses after values for only soft tissue

**Taxon**	**Cell**	**Season**	**Energy ± SD (kJ/g)**
**Annelida**			
** Polychaeta**			
* Unidentified*	1, 3, 5	All	3.59 ± 0.06
* Unidentified*	11	Late	2.42 ± 0.07
** Sipunculida**			
* Unidentified*	11	Late	1.74
**Chordata**			
** Actinopterygii**			
* Ammodytes hexapterus*	1, 3, 5	Early	5.58 ± 0.09
* A. hexapterus*	3	Mid	4.63 ± 0.04
* A. hexapterus*	5	Mid	4.78 ± 0.02
* A. hexapterus*	5	Mid	4.43 ± 0.02
**Cnidaria**			
** Actiniaria**			
* Actinia* spp.	11	Late	2.12 ± 0.08
* Actinia* spp.	11	Late	3.51 ± 0.11
**Crustacea**			
** Amphipoda**			
* Amphipoda* spp.	1	Mid	5.98 ± 0.03
* Amphipoda* spp.	1	Mid	4.71 ± 0.05
* Amphipoda* spp.	3	Late	5.22 ± 0.05
* Amphipoda* spp.	5	Late	4.71 ± 0.03
* Amphipoda* spp.	11	Late	8.11 ± 0.06
* Amphipoda* spp.	11	Late	8.44 ± 0.07
* Amphipoda* spp.	1, 3, 5	Late	4.93 ± 0.04
* Anisogammarus pugettensis*	Unk	Early	4.00 ± 0.01
* Anonyx nugax*	1	Early	3.30 ± 0.08
* A. nugax*, *Grandifoxus robusts*, *Monoporeia affinis*, *Eogammarus schmidti*	1, 3, 5	Early	5.38 ± 0.21
* E. schmidti*, *Eohastorious* spp., *A. pugettensis*	1, 3, 5	Mid	7.35 ± 0.07
* Eohostorious setulogus*	3	Early	3.16
* M. affinis*	1	Early	6.76 ± 0.09
* M. affinis*	3	Early	7.38 ± 0.07
* M. affinis*	3	Early	7.62 ± 0.07
* M. affinis*	5	Early	7.30 ± 0.08
* M. affinis*	5	Early	6.88 ± 0.01
** Cumacea**			
* Unidentified*	1, 3, 5	Unk	4.36 ± 0.03
** Decapoda**			
* Unidentified*	11	Late	4.05 ± 0.33
** Isopoda**			
* Synidotea cinerea*	1, 3	Early	2.97 ± 0.21
* S. cinerea*	1, 3	Mid	3.61 ± 0.18
* S. cinerea*	5	All	3.17 ± 0.00
** Mysida**			
* Unidentified*	1, 3, 5	All	3.34
**Mollusca**			
** Bivalvia, soft tissue only (with shell)**			
* Siliqua alta*	1	Early	5.27 ± 0.08 (3.05 ± 0.05)
* S. alta*	1	Mid	4.97 (2.82)
* S. alta*, *Peronidia*, *unidentified*	1	Mid	3.96 ± 0.05 (2.26 ± 0.03)
* S. alta*, *unidentified*	3	Late	4.11 ± 0.22 (3.33 ± 0.18)
* Unidentified*	1	Late	4.40 ± 0.06 (1.11 ± 0.04)
* Unidentified*	3	Mid	4.65 ± 0.01 (2.90 ± 0.01)
* Unidentified*	5	Early	5.20 ± 0.04 (3.18 ± 0.03)
* Unidentified*	5	Mid	4.72 ± 0.08 (2.89 ± 0.05)
* Unidentified*	5	Late	4.14 ± 0.06 (1.72 ± 0.03)
* Unidentified*	11	Late	4.01 ± 0.14 (3.05 ± 0.05)
**Nemertea**			
** Nemertina**			
* Unidentified*	1	Late	3.1

Moisture content varied among taxa, ranging from 64.4% WM (Isopoda, S*ynidotea cinerea*) to 85.4% WM (Cnidaria, *Actinia spp.*) (Table 2). Mean energy density varied almost seven-fold among benthic taxa, ranging between 1.11 kJ g^−1^ WM (Bivalvia with shell, unidentified spp.) to 7.62 kJ g^−1^ WM (Amphipoda, *Monoporeia affinis*). Amphipods had the highest mean energy density of all prey groups (DM and WM), although there was considerable variation within most groups (Tables 1 and 2). Actinopterygii and bivalves (without shell) also had relatively high energy densities compared to the remaining prey groups.

**Table 2:** The number of samples, percentage water content, and mean and standard deviation (SD) of dry mass (kJ g^−1^ dry mass ± SD) and wet mass (kJ g^−1^ wet mass ± SD) energy density (ED) of the 11 benthic faunal groups collected from NE Sakhalin Island in 2015. Data without SD values indicate sample was too small for replicate measurements

**Taxon**	**Number samples**	**% Water**	**ED (kJ g**^**−1**^** DM)**	**ED (kJ g**^**−1**^** WM)**
**Annelida**				
Polychaeta	2	81.5 ± 2.7	16.10 ± 2.16	3.01 ± 0.83
Sipunculida	1	83.8	10.74	1.74
**Chordata**	4	74.9 ± 2.1	19.40 ± 0.29	4.88 ± 0.48
**Cnidaria**	2	85.4 ± 4.9	19.24 ± 0.28	2.82 ± 0.99
**Crustacea**				
Amphipoda	17	72.5 ± 4.3	20.02 ± 3.67	5.58 ± 1.44
Cumacea	1	64.9	12.42	4.36
Decapoda	1	75.6	16.57	4.05
Isopoda	3	64.4 ± 10.0	10.55 ± 1.38	3.24 ± 0.33
Mysida	1	81.3	17.85	3.34
**Mollusca**				
Bivalvia, soft tissue	10	75.6 ± 1.9	18.46 ± 1.13	4.54 ± 0.49
Bivalvia, with shell	9			2.60 ± 0.76
**Nemertea**	1	80.2	15.68	3.10

**Fig. 2:**
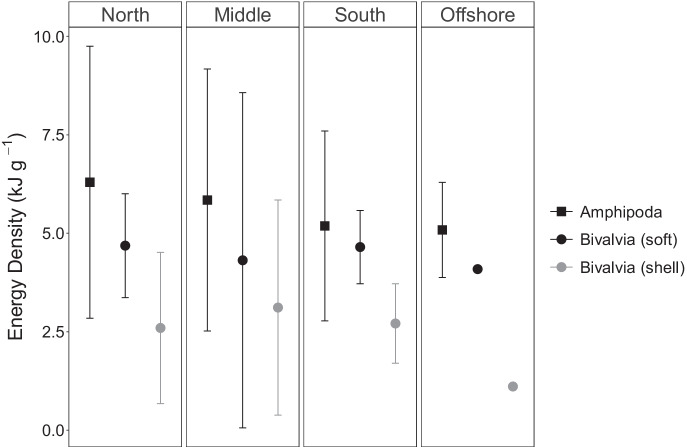
Averages and 95% confidence intervals of Amphipoda and Bivalvia (soft tissue only and with shell) energy density pooled across all locations in the nearshore Piltun feeding area (grouped by “Early,” “Mid,” and “Late” season) compared to the offshore samples, which were only collected during the “Late” sampling period (see text). Sample sizes: nearshore early: Amphipoda *N* = 9, Bivalvia *N* = 2; nearshore mid: Amphipoda *N* = 3, Bivalvia *N* = 3; nearshore late: Amphipoda *N* = 3, Bivalvia *N* = 3; offshore late: Amphipoda *N* = 2, Bivalvia *N* = 1

**Fig. 3:**
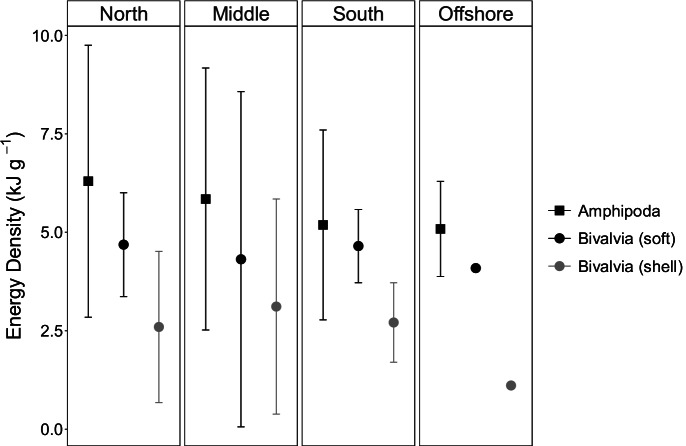
Averages and 95% confidence intervals of Amphipoda and Bivalvia (soft tissue only and with shell) energy density pooled across all seasons in the Piltun feeding area (grouped by north (cell 5), middle (cell 3), and south (cell 1)) and the offshore feeding area (cell 11). Sample sizes: nearshore north: Amphipoda *N* = 6, Bivalvia *N* = 3; nearshore middle: Amphipoda *N* = 7, Bivalvia *N* = 2; nearshore south: Amphipoda *N* = 7, Bivalvia *N* = 4; offshore: Amphipoda *N* = 2, Bivalvia *N* = 1

For the nearshore feeding areas, no differences in average energy densities for amphipods or bivalves were noted across sampling periods (early, mid, and late seasons; Fig. 2) or sampling locations (north, middle, and south; Fig. 3). Similarly, no differences were noted in the energy densities for amphipods or bivalves between the nearshore and offshore feeding areas (Figs. 2 and 3).

### Total energy availability

Biomass sample sizes in cells 1, 3, and 5 were large enough to estimate total energy availability by period. Other cells were sampled less frequently, so biomass samples over the entire foraging season were combined (Table 3). When prey-energy density values were combined with estimates of prey biomass across seasons and foraging locations, the total energy available to foraging gray whales (kJ/m^2^) was higher in the offshore (cells 8–11) than in the nearshore feeding area. Amphipods generally made up a higher proportion of energy in the offshore feeding area than in the nearshore feeding area (Table 3). In addition to shifts in species composition, the mean biomass in the offshore feeding area was higher and, in some cases, an order of magnitude higher than the mean biomass in the nearshore feeding area (Table 3).

**Table 3:** Sample size, total biomass, and total energy content of six prey groups (Actinopterygii, Amphipoda, Bivalvia, Cumacea, Isopoda, and Polychaeta) collected via benthic grabs across seasons. The study area was subdivided into cell numbers as described in the text (Fig. 1b), and grabs were pooled across all sampling stations within a cell and season. The cells comprising the offshore site are emphasized in bold. No grab had zero biomass. Values are means ± standard deviation from multiple samples

**Season**	**Cell**	**Number of biomass grabs**	**Total biomass (six prey) (g/m**^**2**^**)**	**Total energy content (six prey) (kJ/m**^**2**^**)**	**Proportion energy from amphipods**
Early	1	45	110 ± 80	320 ± 200	0.45 ± 0.32
	3	96	110 ± 70	400 ± 240	0.50 ± 0.26
	5	60	110 ± 90	410 ± 270	0.43 ± 0.27
Mid	1	63	70 ± 60	240 ± 170	0.53 ± 0.35
	3	123	80 ± 50	360 ± 280	0.57 ± 0.28
	5	129	120 ± 90	500 ± 330	0.37 ± 0.28
Late	1	36	70 ± 40	220 ± 130	0.33 ± 0.35
	3	63	100 ± 50	400 ± 260	0.54 ± 0.31
	5	63	110 ± 120	340 ± 300	0.47 ± 0.31
All	1	144	80 ± 60	260 ± 180	0.47 ± 0.35
	3	282	90 ± 60	380 ± 260	0.54 ± 0.28
	4	18	60 ± 60	240 ± 230	0.22 ± 0.30
	5	252	120 ± 100	430 ± 320	0.41 ± 0.29
	6	36	70 ± 110	280 ± 380	0.26 ± 0.30
	7	45	160 ± 170	490 ± 540	0.10 ± 0.16
	**8**	**36**	**260 ± 190**	**1030 ± 960**	**0.30 ± 0.23**
	**9**	**39**	**230 ± 210**	**960 ± 930**	**0.37 ± 0.29**
	**10**	**39**	**600 ± 600**	**1900 ± 1560**	**0.28 ± 0.30**
	**11**	**54**	**660 ± 460**	**3310 ± 2540**	**0.77 ± 0.27**

## Discussion

Information on both the biomass and the energy density of prey are necessary for determining the total amount of energy available to western gray whales foraging in the benthos near NE Sakhalin Island, Russia. This knowledge is particularly important if anthropogenic activities are displacing the endangered whales to lower energy regions. While the biomass of the zoobenthos in both the nearshore and offshore feeding areas has been well described, energy density estimates were not available prior to this study.

The diet of gray whales foraging near Sakhalin Island primarily includes species from Actinopterygii (mostly sand lance), Amphipoda, Bivalvia, Cumacea, Isopoda, and Polychaeta (Blanchard et al., 2019). Sakhalin Island amphipods, a particularly important component of the whales’ diet (Demchenko, 2010; Demchenko et al., 2016), were highest in energy density compared to the other prey groups of this area (Tables 1 and 2). This is not surprising given the well-documented energy storage strategy of amphipods such as *M. affinis*, which accumulate energy-rich lipid reserves in anticipation of long periods of food scarcity (e.g., Lehtonen, 1996). Sakhalin Island amphipod values were consistent with those of amphipods from cold-water environments elsewhere: dry mass energy density (20 kJ g^−1^) was on the same order as that of *Monoporeia affinis* from the Baltic (~ 22–28 kJ g^−1^, Lehtonen, 1996) as well as that of Arctic amphipods (~ 17 kJ g^−1^, Highsmith & Coyle, 1992; ~ 19 kJ g^−1^, Hondolero et al., 2012; ~ 16.5–28 kJ g^−1^, Tu et al., 2015). Bivalve shell-free dry mass energy density (~ 18.5 kJ g^−1^) was likewise similar to that seen in other bivalves from northern cold-water systems (~ 19 kJ g^−1^; Brey et al., 1988; Hondolero et al., 2012; Tu et al., 2015), as was energy density for other species where matching data are available (Hondolero et al., 2012; Tu et al., 2015; Wilt et al., 2014).

Amphipods and bivalves sampled late in the season from the offshore feeding area appeared similar in energy density to those sampled nearshore, whether pooled by location (Fig. 2) or season (Fig. 3), although our dataset was insufficient for statistically determining temporal or spatial differences for these or any prey group. However, we were able to test for spatial differences in total prey-energy available (kJ/m^2^) to foraging gray whales by combining energy density data from this study with detailed biomass data reported in separate, complementary studies (Blanchard et al., 2019, 2022). These studies show that, within the nearshore feeding area, overall benthic prey biomass is highest in the middle nearshore area (cell 3) mid-season, when whale abundance in the nearshore area peaks. Late in the foraging season, when gray whale numbers are declining in the nearshore feeding area and increasing in the offshore feeding area, overall benthic prey biomass is generally higher offshore, often substantially so, compared to any of the cells within the nearshore feeding area (Table 3; Blanchard et al., 2019). Our combined datasets show that total energy availability in 2015 was higher in the offshore feeding area compared to the nearshore feeding area as a result of increases in both the absolute and relative numbers of amphipods specifically, rather than higher energy densities of any particular prey group. As a result, Sakhalin Island amphipods make up a substantial proportion of the overall prey-energy available to foraging gray whales across their feeding grounds (Table 3). Amphipods appear to be particularly important in the southwestern-most quadrant of the offshore sampling grid (cell 11), accounting for up to 77% of total prey-energy available.

According to Demchenko and Fadeev (2011), both gray whale feeding areas are numerically dominated by amphipods, though distinct benthic communities characterize the nearshore and offshore sites based on differences in water temperature, water salinity, and sediment type. *Monoporeia affinis* populations dominate benthic communities in the fine-sand habitats nearshore, where high biomass is supported by spring and summer primary production settling on the shallow bottom (Sorokin & Sorokin, 2002). In the fine-to-medium-sand habitats of the offshore feeding area, benthic communities are dominated by tube-dwelling *Ampelisca eschrichtii*, which aggregate in the highest known biomass of amphipods in the world (Demchenko et al., 2016). As these communities occur at depths below the reach of summer primary productivity fluxes, the very high biomass of amphipods in the offshore site is likely supported by lipid reserves built during the winter when oceanographic conditions favor enrichment of the benthos (explained more thoroughly in Blanchard et al., 2019; Demchenko et al., 2016; Durkina et al., 2018). Amphipod biomass off the Sakhalin coast has been declining over the past 15 years, probably due to a combination of ecosystem-level climatic changes that affect local oceanographic processes (e.g., circulation patterns, nutrient supply, seawater temperature, and salinity) and more direct anthropogenic stressors that affect the water quality (e.g., pollution, hypoxia) (Blanchard et al., 2019). The overall energy available across the Okhotsk Sea to western gray whales is thus likely to change, both regionally and basin-wide.

It is worth addressing the management implications of our finding that the offshore feeding area near Sakhalin Island tends to be considerably more energy-rich than the nearshore feeding area. From the perspective of maximizing energy intake, it appears that if ensonified whales were displaced from the nearshore feeding areas in 2015, they would have had access to similar or higher total prey-energy offshore; however, other considerations beyond feeding efficiency contribute to the importance of the nearshore feeding areas to western gray whales. While adult whales, including pregnant and post-weaning females, often feed offshore in late summer, mother and calf pairs as well as weaned calves stay within < 1 km from shore in water depths < 11 m (Schwarz et al., 2022a; Sychenko, 2012). Gray whale mothers, dependent calves and weaned calves may prefer shallower waters close to shore where exposure to marine predators is reduced, and/or where oceanographic conditions are more favorable for young animals.

Disruption of the feeding behaviors of reproductive females in the nearshore area could also affect population growth rates if the offshore area is not a viable foraging alternative for displaced individuals. Gray whales rely on endogenous energy reserves (i.e., fat stores) acquired on their feeding grounds to sustain them through the breeding season, and as little as a 3–4% reduction in annual energy intake may be enough to impact calf production in reproductive females (Villegas-Amtmann et al., 2015, 2017). To explore this idea further, results from this study will be incorporated into energetics models to determine if prey-energy availability within the feeding grounds off Sakhalin Island limits the reproduction or survival of mature females, thereby limiting overall population growth in western gray whales (McHuron et al., 2021; Schwarz et al., 2022b).

## Conclusions

The northeastern Sakhalin Island coast is a region characterized by overlap of essential western gray whale habitat with sites of high anthropogenic activity. Despite multiple stressors to the population, western gray whale numbers have recently started increasing, prompting the IUCN to downlist the whales from “critically endangered” to “endangered” in 2018 (Cooke et al., 2018). This promising development is likely due to the collaborative efforts of conservation groups, scientists, governments, resource extraction industries, and the public. However, food limitation in the region and/or changes in energy balance for individual whales displaced by ensonification may be contributing to the low reproductive rates still limiting population growth. In support of efforts to understand this better, our findings establish an important link between gray whale energy requirements and the energy available to them in their feeding grounds. We show that the energy available in western gray whale prey is similar to that observed in other cold-water environments (Highsmith & Coyle, 1992), and that the offshore feeding area is particularly energy-rich compared to the nearshore feeding area (958–3313 kJ/m^2^ and 223–495 kJ/m^2^, respectively). While less energy-rich, the nearshore feeding area is essential for meeting the energy and safety requirements of mothers and calves. Ecosystem-level changes occurring in the region—including long-term declines in populations of energy-rich amphipods—indicate that assessments of gray whale prey abundance and energy content, as well as the energetic consequences of whale displacement, may be warranted if seismic surveys are to occur in close proximity to the nearshore feeding area.

## Supplementary Information

Below is the link to the electronic supplementary material.

Supplementary file1 (DOCX 30 KB)
