# Supplementary material for: Benthic studies adjacent to Sakhalin Island, Russia, 2015 II: energy content of the zoobenthos in western gray whale feeding grounds
Source: Environ Monit Assess. 2022 Oct 18;194(Suppl 1):742. doi: 10.1007/s10661-022-10020-z (PMC9579061; doi:10.1007/s10661-022-10020-z)
Supplement: Supplementary file 1 — Supplementary file1 (DOCX 30 KB) [file 10661_2022_10020_MOESM1_ESM.docx]

**Supplementary Tables**

**Table S.1** Energy density data pulled from the literature (sources 1-4) for five prey species groups: Amphipoda, Cumacea, Isopoda, Bivalvia, and Polychaeta. The species group Arthropods includes amphipods, cumacea, and isopods. These data were combined with energy density data collected in 2015 for the current study to estimate the total energy available within the gray whale foraging grounds near Sakhalin Island

| **Species group** | **Name** | **Mean** | **StDev** | **Metric** | **Source** |
| --- | --- | --- | --- | --- | --- |
| Actinopterygii | *Ammodytes hexapterus* | 5.79 | 0.54 | kJ/g wet mass | 1 |
| Actinopterygii | *Ammodytes hexapterus* | 5.59 | 0.09 | kJ/g wet mass | 1 |
| Actinopterygii | *Ammodytes hexapterus* | 5.91 | 0.07 | kJ/g wet mass | 1 |
| Actinopterygii | *Ammodytes hexapterus* | 5.71 | 0.12 | kJ/g wet mass | 1 |
| Actinopterygii | *Ammodytes hexapterus* | 5.25 | 0.73 | kJ/g wet mass | 1 |
| Actinopterygii | *Ammodytes hexapterus* | 5.43 | 0.09 | kJ/g wet mass | 1 |
| Actinopterygii | *Ammodytes hexapterus* | 5.38 | 0.15 | kJ/g wet mass | 1 |
| Actinopterygii | *Ammodytes hexapterus* | 4.65 | 0.08 | kJ/g wet mass | 1 |
| Amphipoda | N/A | 1058 | - | cal/g wet mass | 2 |
| Annelida | *Pherusa plumosa* | 463 | - | cal/g wet mass | 2 |
| Annelida | *Aphrodita hastala* | 486 | - | cal/g wet mass | 2 |
| Annelida | *Lumbrineris fragilis* | 1059 | - | cal/g wet mass | 2 |
| Annelida | Maldanidae *(Axiothella, Niochamache)* | 653 | - | cal/g wet mass | 2 |
| Annelida | *Nephthys ciliata* | 747 | - | cal/g wet mass | 2 |
| Annelida | *Pectinaria hyperborea* | 624 | - | cal/g wet mass | 2 |
| Annelida | *Phascolion strombi* | 595 | - | cal/g wet mass | 2 |
| Annelida | *Sternapsis fossor* | 538 | - | cal/g wet mass | 2 |
| Annelida | Terebellidae | 805 | - | cal/g wet mass | 2 |
| Arthropods | *Ampelisca macrocephala* | 4.47 | 0.18 | kcal/g dry mass | 3 |
| Arthropods | *Ampelisca* spp. | 11.41 | – | kJ/g wet mass | 4 |
| Arthropods | Amphipoda spp. | 5.22 | 0.24 | kcal/g dry mass | 5 |
| Arthropods | *Anonyx* spp. | 7.76 | 0.07 | kJ/g wet mass | 4 |
| Arthropods | *Argis lar* | 5.43 | 0.27 | kJ/g wet mass | 4 |
| Arthropods | *Argis lar* | 4.47 | 0.26 | kcal/g dry mass | 3 |
| Arthropods | *Argis lar* | 4.86 | 0.25 | kcal/g dry mass | 3 |
| Arthropods | *Argis lar* | 5.1 | 0.2 | kcal/g dry mass | 3 |
| Arthropods | *Byblis gaimardi* | 4.48 | – | kcal/g dry mass | 3 |
| Arthropods | *Chionoecetes opilio* | 4.97 | 0.32 | kJ/g wet mass | 4 |
| Arthropods | *Hyas coarctatus* | 2.58 | 0.13 | kJ/g wet mass | 4 |
| Arthropods | *Hyas coarctatus* | 3.39 | 0.69 | kcal/g dry mass | 3 |
| Arthropods | *Hyas coarctatus* | 3.63 | – | kcal/g dry mass | 3 |
| Arthropods | *Hyas coarctatus* | 3.7 | – | kcal/g dry mass | 3 |
| Arthropods | *Hyas coarctatus* | 4.38 | – | kcal/g dry mass | 3 |
| Arthropods | *Hyas coarctatus* | 5.12 | – | kcal/g dry mass | 3 |
| Arthropods | Idoteidae | 17.72 | – | MJ/kg dry mass | 6 |
| Arthropods | Lysianassidae | 19.48 | – | MJ/kg dry mass | 6 |
| Arthropods | Mysidacea | 17.4 | – | MJ/kg dry mass | 6 |
| Arthropods | Mysidacea | 21.61 | – | MJ/kg dry mass | 6 |
| Arthropods | Mysidacea | 21.44 | – | MJ/kg dry mass | 6 |
| Arthropods | Mysidacea | 23.24 | – | MJ/kg dry mass | 6 |
| Arthropods | Mysidacea | 19.63 | – | MJ/kg dry mass | 6 |
| Arthropods | Mysidacea | 22.29 | – | MJ/kg dry mass | 6 |
| Arthropods | Mysidacea | 19.68 | – | MJ/kg dry mass | 6 |
| Arthropods | Mysidacea | 19.55 | – | MJ/kg dry mass | 6 |
| Arthropods | Oregoniidae | 14.95 | – | MJ/kg dry mass | 6 |
| Arthropods | Oregoniidae | 15.28 | – | MJ/kg dry mass | 6 |
| Arthropods | Oregoniidae | 15.67 | – | MJ/kg dry mass | 6 |
| Arthropods | Oregoniidae | 16.06 | – | MJ/kg dry mass | 6 |
| Arthropods | Oregoniidae | 17.64 | – | MJ/kg dry mass | 6 |
| Arthropods | Oregoniidae | 18.83 | – | MJ/kg dry mass | 6 |
| Arthropods | Oregoniidae | 18.7 | – | MJ/kg dry mass | 6 |
| Arthropods | Oregoniidae | 18.81 | – | MJ/kg dry mass | 6 |
| Arthropods | Oregoniidae | 17.85 | – | MJ/kg dry mass | 6 |
| Arthropods | Oregoniidae | 20.26 | – | MJ/kg dry mass | 6 |
| Arthropods | Oregoniidae | 17.67 | – | MJ/kg dry mass | 6 |
| Arthropods | Oregoniidae | 19.06 | – | MJ/kg dry mass | 6 |
| Arthropods | Oregoniidae | 17.28 | – | MJ/kg dry mass | 6 |
| Arthropods | Paguridae | 17.28 | – | MJ/kg dry mass | 6 |
| Arthropods | Paguridae | 17.21 | – | MJ/kg dry mass | 6 |
| Arthropods | Paguridae | 17.72 | – | MJ/kg dry mass | 6 |
| Arthropods | Paguridae | 17.9 | – | MJ/kg dry mass | 6 |
| Arthropods | Paguridae | 17.44 | – | MJ/kg dry mass | 6 |
| Arthropods | Paguridae | 17.92 | – | MJ/kg dry mass | 6 |
| Arthropods | Paguridae | 17.88 | – | MJ/kg dry mass | 6 |
| Arthropods | Paguridae | 17.26 | – | MJ/kg dry mass | 6 |
| Arthropods | Paguridae | 18.19 | – | MJ/kg dry mass | 6 |
| Arthropods | Paguridae | 17.72 | – | MJ/kg dry mass | 6 |
| Arthropods | Paguridae | 20.89 | – | MJ/kg dry mass | 6 |
| Arthropods | Paguridae | 20.15 | – | MJ/kg dry mass | 6 |
| Arthropods | *Pagurus* spp. | 6.26 | 0.33 | kJ/g wet mass | 4 |
| Arthropods | *Sclerocrangon* sp. | 4.64 | 0.17 | kcal/g dry mass | 3 |
| Arthropods | *Sclerocrangon* sp. | 4.53 | 0.16 | kcal/g dry mass | 3 |
| Arthropods | *Sclerocrangon* sp. | 4.87 | 0.11 | kcal/g dry mass | 3 |
| Arthropods | *Stegocephalus* spp. | 5.19 | 0.15 | kJ/g wet mass | 4 |
| Bivalves | *Astarte borealis* | 0.74 | – | kJ/g wet mass | 4 |
| Bivalves | *Astarte montagui* | 0.62 | 0.01 | kJ/g wet mass | 4 |
| Bivalves | *Astarte* spp. | 0.71 | 0.01 | kJ/g wet mass | 4 |
| Bivalves | Astartidae | 21.15 | – | MJ/kg dry mass | 6 |
| Bivalves | Astartidae | 17.6 | – | MJ/kg dry mass | 6 |
| Bivalves | Astartidae | 21.42 | – | MJ/kg dry mass | 6 |
| Bivalves | Astartidae | 22.25 | – | MJ/kg dry mass | 6 |
| Bivalves | Astartidae | 19.95 | – | MJ/kg dry mass | 6 |
| Bivalves | Astartidae | 21.21 | – | MJ/kg dry mass | 6 |
| Bivalves | Astartidae | 20.91 | – | MJ/kg dry mass | 6 |
| Bivalves | Astartidae | 21.04 | – | MJ/kg dry mass | 6 |
| Bivalves | Astartidae | 20.09 | – | MJ/kg dry mass | 6 |
| Bivalves | Astartidae | 18.9 | – | MJ/kg dry mass | 6 |
| Bivalves | Cardiidae | 20.22 | – | MJ/kg dry mass | 6 |
| Bivalves | Cardiidae | 20.7 | – | MJ/kg dry mass | 6 |
| Bivalves | Carditidae | 21.88 | – | MJ/kg dry mass | 6 |
| Bivalves | Carditidae | 22.04 | – | MJ/kg dry mass | 6 |
| Bivalves | Carditidae | 21.73 | – | MJ/kg dry mass | 6 |
| Bivalves | Carditidae | 21.13 | – | MJ/kg dry mass | 6 |
| Bivalves | *Ennucula tenuis* | 2.22 | 0.22 | kJ/g wet mass | 4 |
| Bivalves | *Ennucula tenuis* | 4.77 | 0.08 | kcal/g dry mass | 3 |
| Bivalves | *Macoma calcarea* | 4.92 | 0.22 | kcal/g dry mass | 3 |
| Bivalves | *Macoma moesta* | 4.42 | – | kcal/g dry mass | 3 |
| Bivalves | *Macoma moesta* | 4.56 | – | kcal/g dry mass | 3 |
| Bivalves | *Macoma* spp. | 2.23 | 0.17 | kJ/g wet mass | 4 |
| Bivalves | Mytilidae | 19.64 | – | MJ/kg dry mass | 6 |
| Bivalves | Mytilidae | 21.42 | – | MJ/kg dry mass | 6 |
| Bivalves | Mytilidae | 21.32 | – | MJ/kg dry mass | 6 |
| Bivalves | *Nuculana radiata* | 0.98 | 0.47 | kJ/g wet mass | 4 |
| Bivalves | Nuculanidae | 23.23 | – | MJ/kg dry mass | 6 |
| Bivalves | Nuculanidae | 22.06 | – | MJ/kg dry mass | 6 |
| Bivalves | Nuculanidae | 21.87 | – | MJ/kg dry mass | 6 |
| Bivalves | Nuculanidae | 17.91 | – | MJ/kg dry mass | 6 |
| Bivalves | Nuculanidae | 23.49 | – | MJ/kg dry mass | 6 |
| Bivalves | Nuculidae | 21.03 | – | MJ/kg dry mass | 6 |
| Bivalves | Nuculidae | 21.42 | – | MJ/kg dry mass | 6 |
| Bivalves | Nuculidae | 22 | – | MJ/kg dry mass | 6 |
| Bivalves | Nuculidae | 20.83 | – | MJ/kg dry mass | 6 |
| Bivalves | Nuculidae | 20.67 | – | MJ/kg dry mass | 6 |
| Bivalves | Nuculidae | 20.23 | – | MJ/kg dry mass | 6 |
| Bivalves | Nuculidae | 17.63 | – | MJ/kg dry mass | 6 |
| Bivalves | Nuculidae | 20.83 | – | MJ/kg dry mass | 6 |
| Bivalves | Nuculidae | 22.43 | – | MJ/kg dry mass | 6 |
| Bivalves | *Serripes groenlandicus* | 5.45 | 0.19 | kcal/g dry mass | 3 |
| Bivalves | Tellinidae | 20.02 | – | MJ/kg dry mass | 6 |
| Bivalves | Tellinidae | 21.87 | – | MJ/kg dry mass | 6 |
| Bivalves | Tellinidae | 21.46 | – | MJ/kg dry mass | 6 |
| Bivalves | Tellinidae | 21.62 | – | MJ/kg dry mass | 6 |
| Bivalves | Tellinidae | 22.76 | – | MJ/kg dry mass | 6 |
| Bivalves | Tellinidae | 20.78 | – | MJ/kg dry mass | 6 |
| Bivalves | Tellinidae | 22.56 | – | MJ/kg dry mass | 6 |
| Bivalves | Tellinidae | 18.8 | – | MJ/kg dry mass | 6 |
| Bivalves | Tellinidae | 22.67 | – | MJ/kg dry mass | 6 |
| Bivalves | Tellinidae | 21.74 | – | MJ/kg dry mass | 6 |
| Bivalves | Various | 4.85 | 0.13 | kcal/g dry mass | 5 |
| Bivalves | Veneridae | 20.77 | – | MJ/kg dry mass | 6 |
| Bivalves | Veneridae | 20.05 | – | MJ/kg dry mass | 6 |
| Bivalves | *Yoldia hyperborea* | 2.26 | – | kJ/g wet mass | 4 |
| Bivalves | Yoldiidae | 21.77 | – | MJ/kg dry mass | 6 |
| Bivalves | Yoldiidae | 20.84 | – | MJ/kg dry mass | 6 |
| Bivalves | Yoldiidae | 21.57 | – | MJ/kg dry mass | 6 |
| Bivalvia |  |  |  |  |  |
| - whole organism | *Clinocardium ciliatum* | 374 | - | cal/g wet mass | 2 |
| Bivalvia |  |  |  |  |  |
| - whole organism | *Yoldia thraciaeformis* | 509 | - | cal/g wet mass | 2 |
| Bivalvia |  |  |  |  |  |
| - whole organism | *Yoldia sapotilla* | 688 | - | cal/g wet mass | 2 |
| Decapoda | *Argis dentata*, mixed adults | 1081 | - | cal/g wet mass | 2 |
| Decapoda | *Argis dentata* 9 with eggs | 1158 | - | cal/g wet mass | 2 |
| Decapoda | *Pandalus montagui* | 1320 | - | cal/g wet mass | 2 |
| Echinodermata | *Ctenoiliscus crispatus* | 608 | - | cal/g wet mass | 2 |
| Echinodermata | *Asterias vulgaris* | 633 | - | cal/g wet mass | 2 |
| Polychaeta | *Cistenides hyperborea* | 2.45 | – | kcal/g dry mass | 3 |
| Polychaeta | *Cistenides hyperborea* | 2.55 | – | kcal/g dry mass | 3 |
| Polychaeta | Lumbrineridae | 20.52 | – | MJ/kg dry mass | 6 |
| Polychaeta | *Lumbrineris* spp. | 5.42 | 0.07 | kJ/g wet mass | 4 |
| Polychaeta | Maldanidae | 19 | – | MJ/kg dry mass | 6 |
| Polychaeta | Maldanidae | 20.24 | – | MJ/kg dry mass | 6 |
| Polychaeta | Maldanidae | 20.68 | – | MJ/kg dry mass | 6 |
| Polychaeta | Maldanidae | 20.54 | – | MJ/kg dry mass | 6 |
| Polychaeta | Maldanidae | 20.34 | – | MJ/kg dry mass | 6 |
| Polychaeta | Maldanidae | 19.21 | – | MJ/kg dry mass | 6 |
| Polychaeta | Maldanidae | 21.2 | – | MJ/kg dry mass | 6 |
| Polychaeta | Maldanidae | 21.61 | – | MJ/kg dry mass | 6 |
| Polychaeta | Maldanidae | 21.34 | – | MJ/kg dry mass | 6 |
| Polychaeta | Maldanidae | 3.41 | 0.06 | kJ/g wet mass | 4 |
| Polychaeta | Nephtyidae | 20.25 | – | MJ/kg dry mass | 6 |
| Polychaeta | Nephtyidae | 21.86 | – | MJ/kg dry mass | 6 |
| Polychaeta | *Nephtys* spp. | 4.49 | – | kJ/g wet mass | 4 |
| Polychaeta | Nepthydidae | 3.77 | – | kcal/g dry mass | 3 |
| Polychaeta | Onuphidae | 20.6 | – | MJ/kg dry mass | 6 |
| Polychaeta | *Paradiopatra parva* | 3.34 | – | kJ/g wet mass | 4 |
| Polychaeta | *Paradiopatra* spp. | 5.58 | – | kJ/g wet mass | 4 |
| Polychaeta | Phyllodocidae | 20.25 | – | MJ/kg dry mass | 6 |
| Polychaeta | Polynoidae | 19.77 | – | MJ/kg dry mass | 6 |
| Polychaeta | Polynoidae | 2.84 | 0.01 | kJ/g wet mass | 4 |
| Polychaeta | Terebellidae | 3.6 | 0.08 | kJ/g wet mass | 4 |
| Polychaeta | Various | 3.6 | 0.76 | kcal/g dry mass | 5 |
| Sources: 1. Anthony et al. 2000; 2. Brawn et al. 1968; 3. Hondolero et al. 2012; 4. Tu et al. 2015; 5. Stoker 1978; 6. Wilt et al. 2014 | | | | | |

**Table S.2** Best-fit distributions of energy density for six benthic prey groups. Sampling distributions required rescaling or transformation after sampling, so the units are not consistent across distributions. However, after rescaling or tranforming, all values are in cal/g wet mass

| **Group** | **Distribution** | **Mean** | **SD** | **Min** | **Max** | **N** |
| --- | --- | --- | --- | --- | --- | --- |
| Amphipoda | lognormal^*^ | 7.2 | 0.3 | 4.8 | 8.3 | 29 |
| Bivalvia | normal | 498.9 | 211.9 | 0 | 797 | 19 |
| Actinopterygii | lognormal^*^ | 7.1 | 0.1 | 6.2 | 7.6 | 12 |
| Polychaeta | normal | 1003.9 | 345.6 | 103.7 | 2649.9 | 46 |
| Cumacea | lognormal^*^ | 7.1 | 0.4 | 5.3 | 8.3 | 1^†^ |
| Isopoda | scaled beta^‡^ |  |  |  |  | 7 |
|  |  |  |  |  |  |  |
| ^†^+67 Decapoda,Isopoda,Mysida | | | | | | |
| ^‡^alpha = 3.49, beta = 9.10, min = 0.04, max = 1.00, scale = 4104.60+0.1 | | | | | | |
| ^*^ For sampling, sample from normal with given mean and standard deviation, then take the exponent. | | | | | | |
